# Reactive and Regulative Temperament in Relation to Clinical Symptomatology and Personality Disorders in Patients with a Substance Use Disorder

**DOI:** 10.3390/jcm11030591

**Published:** 2022-01-25

**Authors:** Els Santens, Geert Dom, Eva Dierckx, Laurence Claes

**Affiliations:** 1 Alexian Psychiatric Hospital, 3300 Tienen, Belgium; eva.dierckx@azt.broedersvanliefde.be; 2 Faculty of Medicine and Health Sciences, University of Antwerp, 2610 Antwerp, Belgium; geert.dom@uantwerpen.be (G.D.); laurence.claes@uantwerpen.be (L.C.); 3 Multiversum Psychiatric Hospital, 2530 Boechout, Belgium; 4 Developmental and Life Span Psychology, Vrije Universiteit Brussel, 1050 Brussels, Belgium; 5 Faculty of Psychology and Educational Science, Katholieke Universiteit Leuven, 3000 Leuven, Belgium

**Keywords:** substance use disorders, effortful control, behavioral inhibition, behavioral activation, clinical symptoms, personality disorders

## Abstract

Temperament and personality traits are important factors underlying the vulnerability for both the initiation and continuation of addictive behaviors. We investigated the influence of reactive and regulative temperament and their interaction in relation to clinical symptomatology and personality disorders (PDs) in a sample of 841 inpatients (68.1% males) with a substance use disorder (SUD). To assess reactive temperament we used the Behavioral Inhibition and Behavioral Activation Scales (BISBAS) and to assess regulative temperament we used the Effortful Control Scale. Clinical symptomatology and personality traits were measured by means of the Symptom Checklist-90 (SCL-90) and the Assessment of ADP-IV Personality Disorders (ADP-IV). Hierarchical regression analyses showed that both, clinical symptomatology and PDs were related to low levels of effortful control (EC). None of the two-way interactions (BIS × EC, BAS × EC) however were significantly related to psychopathology. Current findings highlight the role of effortful control (EC) in the expression of psychopathology in an adult sample of inpatients with SUD. Therapeutic interventions aiming at strengthening EC can possibly result in better treatment outcomes for both the addiction and the comorbid psychopathology.

## 1. Introduction

SUDs are highly prevalent disorders with reported lifetime prevalence rates of any substance abuse or dependence between 10–20% in the general population [1,2] and constitute a major public health problem [3]. SUDs are heterogeneous disorders characterized by compulsive drug seeking/taking, the inability to limit intake and the experience of negative affect and withdrawal symptoms in absence of substances [4]. A vulnerability to disinhibition or a lack of self-regulation seems to be a core risk factor associated with both the initiation and continuation of substance use disorders [5]. SUDs frequently co-occur with other psychiatric disorders such as mood and anxiety disorders [6], personality disorders (PDs) and psychotic disorders [7,8].

Self-regulation or EC refers to the ability to regulate behaviors, emotions and cognitions. In a review by Santens et al. (2020) EC is considered as a transdiagnostic dimension underlying externalizing (e.g., SUDs, ADHD) as well as internalizing (e.g., anxiety and mood disorders) psychopathology [9]. EC is a regulative dimension of temperament that involves attentional control, inhibitory control and activation control, and reflects self-regulation abilities that develop later in life parallel with the maturation of the prefrontal cortex and refers to top-down control [10]. According to the dual pathways model, psychopathology arises from an imbalance between two complementary neurobiological systems: (1) the impulsive system or the bottom-up reactivity in terms of behavioral inhibition (BIS) and behavioral activation (BAS) system and (2) the reflective system or top-down regulation in terms of EC [11,12,13]. Vulnerability theories of psychopathology emphasize the role of self-regulation or EC which may moderate the association between temperamental (BISBAS) reactivity and psychopathology [8,9,14,15,16,17,18].

The reactive dimensions of temperament are described in Gray’s Reinforcement Sensitivity Theory (RST), in which BAS refers to sensitivity to reward and BIS to sensitivity to punishment [13]. These reactive temperamental traits defined by the RST refer to bottom-up processes which can already be observed in early childhood. Overactivation of BIS or BAS reactivity can be related to different forms of psychopathology. People with high BAS activation tend to be more impulsive and extraverted, whereas higher BIS activation results in higher proneness to anxiety and is associated with Neuroticism [19,20,21]. Additionally, internalizing problems (e.g., anxiety and mood disorders) are more related to an overactive BIS, whereas externalizing problems (e.g., SUDs) are related to an overactive BAS and an underactive BIS which fails to inhibit inappropriate behaviour that was initiated by BAS [22,23]. Clinical research points out that individuals with low levels of EC are at increased risk for multiple types of psychopathologies see [8]. In terms of reactive and regulative temperament, EC often moderates the relationship between BISBAS reactivity and psychopathology: internalizing disorders (e.g., anxiety and mood disorders) more often seem to be characterized by high levels of BIS and low levels of EC, whereas externalizing disorders (e.g., SUDs, ADHD) by high levels of BAS and low levels of EC [8,16].

In terms of both reactive and regulative temperament, we expect SUDs to be especially characterized by high levels of BAS and low levels of EC. In the literature we indeed found BAS sensitivity in particular to be linked to several types of substance abuse and acting as a predictor of reactivity to alcohol cues, cue-elicited craving and positive alcohol expectancies [24,25,26].

Additionally, we know that a lack of self-regulatory processes (low EC) is also a core risk factor for both the initiation and continuation of SUDs [5,27]. Several studies examined the role of EC in SUDs in which low EC was related to SUD at all stages of addiction [28,29]. High EC was linked to less substance use and a lower drinking frequency [30,31]. Chronic use of substances is known to undermine the efficiency of control networks, including dorsolateral prefrontal cortex, ventromedial prefrontal cortex, and anterior cingulate cortex regions, weakening the capacity for self-regulation when exposure to drug cues occurs and worsens the cycle of addiction [30,32].

Importantly previous research in a large clinical sample of SUD patients highlighted the role of BIS as well. It was found that the cluster of SUD patients characterized by high BIS and low EC had the highest levels of psychopathology on all clinical symptoms (especially on internalizing symptoms: depression and anxiety) and more cluster B and C PDs as compared with the cluster of SUD patients characterized by high BAS and low EC [33].

Of importance, these temperamental factors are implicated in a broad spectrum of psychiatric disorders [8]. A sizeable number of patients with SUD have comorbid psychopathologies such as mood and anxiety disorders and PDs (especially the antisocial PD (ASPD) and the borderline PD (BPD) [6,34,35,36]. Further, psychotic disorders and schizophrenia are also highly comorbid with SUDs [1,2,3]. We will further elucidate the role of BIS, BAS and EC underlying the comorbid mood and anxiety disorders and PDs in a large sample of patients with SUD.

Mood and anxiety disorders are the most prevalent clinical disorders in the general population [37]. The report of EMDCCA on comorbidity of SUD and mental disorders in Europe (2015) reports that (a) depression with SUD is the most common comorbidity, with prevalence rates ranging from 12% to 80%, depending on the characteristics of the sample (e.g., clinical versus non-clinical samples, diagnostic criteria used), and that (b) anxiety disorders are also commonly seen in association with SUD, with prevalence rates as high as 35%. However, the causal relationships between anxiety disorders and SUD (self-medication theories, substance-induced anxiety) are not clearly established and also depend on the specific combination of drugs (e.g., cocaine, cannabis) and anxiety disorders (e.g., post-traumatic stress disorder, panic disorder) [38].

Mood and anxiety disorders are marked by high levels of BIS, low levels of BAS (for depression) and low levels of EC [18,39,40,41]. High BIS is assumed to be a vulnerability factor to internalizing pathology [1] and underlies the personality dimension of anxiety. Additionally, research linked depression to a reward hyposensitivity (low BAS) resulting in a lower approach motivation [18]. Furthermore, behavioral activation interventions have played an important role in treating depressive episodes and reducing relapses [42]. Previous results also indicated that low BAS sensitivity is not only a potential marker of vulnerability to depression but also may be useful in predicting the course of the depressive disorder [18,42].

PDs are defined as enduring and maladaptive patterns of perceiving, thinking about, relating to and interacting with people. PDs can be grouped into three clusters: Cluster A is characterized by eccentric/odd behaviour, Cluster B by erratic/dramatic behaviour and Cluster C by anxious/avoidant behaviour [43]. PDs research shows that cluster B PDs were characterized by high BAS, cluster C PDs by high BIS and cluster A PDs by a mixed pattern of BIS/BAS [18]. Furthermore, several studies have described impairments in self-regulation capacities (low EC) in PDs [44,45,46].

PDs and SUDs commonly co-occur with prevalence rates of PDs in patients with SUD ranging from 24% to 90% depending on the sample characteristics and settings [47]. Concerning the high co-occurrence between SUDS and BPD and ASPD, emotion dysregulation as well as impulsivity play an important role in both disorders [48].

There is growing interest in the possible transdiagnostic role of EC in both SUDs and in their comorbid disorders. It would thus be of clinical interest to investigate the influence of reactive (BIS/BAS) and regulative temperament (EC) and their interaction in a large sample of SUD patients in relation to clinical symptomatology and personality disorders providing new insights in understanding the role of temperamental factors developing co-morbid psychological problems with SUDs. Therefore, in the current study, we want to expand existing research by examining whether effortful control moderates the influence of reactive (BIS/BAS) temperament in relation to clinical symptomatology and PDs in a large sample of adult inpatient with SUDs.

Based on the current theoretical perspective that the interaction between certain reactive temperament traits (BIS/BAS) and self-regulatory capacities (EC) may increase or decrease the risk for psychopathology [14,15,16,17,18], we expect to find (a) concerning clinical symptomatology that internalizing symptoms are related to high levels of BIS and externalizing symptoms to be related to high levels of BAS in combination with low EC [18,30] and (b) concerning PDs we expect Cluster B PDs to be related to high levels of BAS and Cluster C PDs to high levels of BIS [6,18,33,34,35,36,44,46] in combination with low EC.

## 2. Materials and Methods

### 2.1. Participants and Procedure

The study included 841 adult Caucasian inpatients (68.1% males, 38.8% females and 0.1 % gender unknown) consecutively admitted from April 2015 till June 2020 at the treatment unit for addiction of the Alexian Psychiatric Hospital, Tienen, Belgium. This unit provides a residential cognitive behavioral therapy program for patients with a SUD.

In this sample, 177 (21%) patients only used alcohol, 583 (69.2%) used alcohol and another substance and 57 (6.8%) patients used another substance, such as amphetamine, cocaine, cannabis, benzodiazepines, and opioid analgetics (Table 1). The SUDs most frequently seen at our treatment service are alcohol use disorders (21.5%), alcohol use disorder and sedative, hypnotic and anxiolytic use disorder (28.8%) and polysubstance use disorder (≥3 substances) (31.9%). Cocaine Use Disorder accounts for 1.2%, Cannabis Use Disorder for 0.6% and Sedative, Hypnotic and Anxiolytic Use Disorder for 3.1%, see Supplementary Materials Table S1.

Experienced psychiatrists assessed the patients by means of the criteria of the Diagnostic and Statistical Manual of Mental Disorders, Fifth Edition (DSM-5, APA, 2013) for SUDs. The mean age of the participants was 42.86 years (*SD* = 11.74, range: 17–71 years). The self-report questionnaires were administered after detoxification during the second week of admission by means of a computer on the ward. All patients signed an informed consent form before participating in the study.

### 2.2. Measures

#### 2.2.1. Behavioral Inhibition System (BAS) and Behavioral Activation System (BAS) Scales (BIS/BAS)

The BIS/BAS scales is a self-report questionnaire consisting of 24 items which are rated on a 4-point Likert scale ranging from ‘1 = I strongly agree’ to ‘4 = I strongly disagree’ which assess the reactivity of the BIS and BAS systems [13]. Cronbach’s alpha coefficient of the BIS scale is 0.79 and of the BAS total scale is 0.85 representing acceptable internal consistency in the present sample.

#### 2.2.2. Adult Temperament Questionnaire Short Form (ATQ-ECS)

The Effortful Control Scale of the ATQ is a self-report scale consisting of 19 items which are rated on a 7-point Likert scale from ‘1 = I entirely agree’ to ‘7 = I entirely disagree’, which measures an individual’s regulatory capacity [49]. The ECS is found to be a reliable measure with good internal consistency and construct validity [50]. The EC total score demonstrated acceptable internal consistency (α = 0.81).

#### 2.2.3. Symptom-Checklist-90-Revised (SCL-90-R, Dutch Version)

The SCL-90-R is a widely used self-report questionnaire for measuring a range of psychological and psychiatric symptoms consisting of 90 items which are rated on a 5-point Likert scale ranging from ‘1 = not at all” to ‘5 = extremely true”. It involves nine primary symptom dimensions: depression (DEP), anxiety (ANX), agoraphobia (AGO), somatization (SOM), insufficiency of thought and behavior (IN), hostility (HOS), sleeping problems (SLE), distrust and interpersonal sensitivity (DIS) experienced in the past 7 days [51]. In the present study, Cronbach’s alphas are the following: DEP = 0.93, ANX = 0.89, AGO = 0.84, SOM = 0.84, INS = 0.84, SEN = 0.90, HOS = 0.79, DIS = 0.78 representing acceptable internal consistency. Several studies have shown high sensitivity and moderate specificity for the SCL-90-R when used as a screening instrument for mental disorders in SUD patients [52].

#### 2.2.4. Assessment of DSM-IV Personality Disorders (ADP-IV)

The ADP-IV consists of 94 items which assesses the risk for 10 DSM-IV PDs [53]. The items are rated on a 7-point Likert scale (trait score) ranging from ‘1 = totally applicable’ to ‘7 = entirely false’. Summing the scores on the “trait” items for their corresponding scale results in dimensional PD scales.

In the present sample Cronbach’s alphas of the ADP-IV dimensional scales range from 0.64 (Schizotypal PD) till 0.86 (Avoidant PD) representing marginally acceptable to acceptable internal consistency coefficients.

#### 2.2.5. Drug Use Screening Inventory-Revised (DUSI-R)

The DUSI-R is a self-report questionnaire which assesses 20 types of substance used the past three months. We divided them into three categories as follows: alcohol use, alcohol use and another substance and use of other substances [54].

### 2.3. Statistical Analyses

Statistical analyses were conducted by means of SPSS Statistics 26. We performed a series of hierarchical regression analyses with the SCL-90 clinical symptom subscales and the ADP-IV-dimensional PD scores as dependent variables and age, gender (step 1), the main effects of BIS, BAS and EC (step 2) and the two-way interactions (BIS × EC, BAS × EC) (step 3) as predictors. The BIS × BAS interaction was not included because we were particularly interested in the interaction of both BIS/BAS and EC. To compute interaction terms, the independent variables were first standardized.

## 3. Results

### 3.1. Characteristics of the Sample

In this sample, 177 (21%) patients only used alcohol, 583 (69.2%) used alcohol and another substance and 57 (6.8%) patients used another substance, such as amphetamine, cocaine, cannabis, benzodiazepines, and opioid analgetics (Table 1 and Supplementary Materials Table S1).

Table 1 and Table 2 set out the characteristics of the sample.

### 3.2. Influence of Reactive and Regulative Temperamental Aspects and Their Interaction in Relation to Clinical Symptomatology in Inpatients with SUD

The results of the hierarchical regression analyses with the SCL-90 symptoms scales as dependent variables are displayed in Table 3.

The results clearly showed that most of the clinical symptoms (anxiety, depression, obsessive compulsive disorder, interpersonal sensitivity, and the total score) were significantly related to high levels of BIS reactivity and low levels of EC. Sleep problems and somatic complaints were related to female gender and low EC; whereas hostility was associated with a younger age and low EC. None of the two-way interactions (BIS × EC, BAS × EC) were significantly related to clinical symptomatology in patients with SUD.

### 3.3. Influence of Reactive and Regulative Temperamental Aspects and Their Interaction in Relation to PDs in Inpatients with SUD

The results of the hierarchical regression analyses with the dimensional ADP-IV as dependent variables are displayed in Table 4.

All PDs were related to low levels of EC.

Further, Cluster B PDs were related to high levels of BAS reactivity for the narcissistic and histrionic PD and cluster C PDs were associated with high levels of BIS reactivity. None of the two-way interaction terms (BIS × EC, BAS × EC) were significantly related to PDs in inpatients with SUD.

## 4. Discussion

In the present study, we investigated the influence of reactive (BIS/BAS) and regulative (EC) temperament and their interaction on clinical symptomatology and PDs in adult inpatients with SUD. In our sample most patients had an AUD (21%) or used alcohol in combination with other substance(s) (68.2%).

Concerning clinical symptomatology, the results clearly showed the most of clinical symptoms (anxiety, depression, OCD, interpersonal sensitivity, and the total score) were associated with high levels of BIS reactivity and low levels of EC. These findings are in line with the existing literature stating that internalizing disorders (e.g., anxiety and mood disorders) are characterized by low levels of EC and high levels of BIS [8,18,33]. In SUDS there is a high comorbidity of mood and anxiety disorders [6,34,55]. A meta-analysis indicated the strongest associations between illicit drug use disorder and major depression, followed by illicit drug use and any anxiety disorder, alcohol use disorders and major depression and alcohol use disorders and any anxiety disorder diagnoses based on lifetime or 12-month prevalence [56].

Drinking to cope with anxiety and negative affectivity is a potent marker for current and future problems with alcohol in high BIS individuals and may point to negative reinforcement drinking [5,57,58]. Research shows evidence for the overlapping neurobiology of negative affect and SUD. The negative affect, associated with withdrawal is linked with a diminished activation in the reward circuitry and activation of the stress neurotransmitters in the extended amygdala. The amygdala is also connected to brain areas involved in executive function (medial prefrontal cortex), emotion regulation, stress reactivity (paraventricular hypothalamus and locus coeruleus), and reward processing (nucleus accumbens shell and ventral tegmental area [55,59,60]. Research also shows that chronic alcohol use results in neuroadaptations to the central amygdala that are similar to the neuroadaptations that occur after chronic stress [55]. Further, research suggests that mood disorders and SUDs are both associated with deficits in the brain reward circuits and memory deficits [61]. Anhedonia and lack of motivation, both symptoms of depression have been linked to dysfunction of the dopaminergic system [62,63]. These findings may explain the high co-occurrence of mood disorders and alcohol use disorder (AUD).

Concerning PDs we found that all PDs were related to low levels of EC; none of the interaction terms were statistically significant.

ASPD is found to be highly associated with SUDs, mainly alcohol, cannabis and tobacco use and seems to reflect a general vulnerability to externalizing behaviors [64,65]. AUD is more severe in patients with a ASPD (earlier age of onset and more rapid progression to dependence [65]. Research also pointed out that “the traits of ASPD, such as deficits in executive function and response regulation as well as anxious-impulsive personality traits may represent endophenotypes associated with greater risk of developing cocaine and amphetamine use disorder” [65]. Studies have shown a prevalence between 30–50% for BPD and SUDs [36]. From a symptom perspective, Cluster B PD symptoms are uniquely associated with AUD [64].The link between the diagnoses SUDs and BPD seems to consist in impulsivity, emotional dysregulation and negative emotionality [65]. Symptoms of delusions of reference and social anxiety are in some studies linked to schizotypal traits (cluster A PD) that could predict cannabis consumption [65].

In sum, our study shows that low EC is involved in all clinical symptomatology and PDs in our sample of inpatients with SUDs; high BIS is related to internalizing symptomatology and cluster C PDs, and high BAS is related to hostility and the narcissistic and histrionic PD of Cluster B PDs. We only found a main effect of both regulative (EC) and reactive (BIS/BAS) temperament but not a moderating effect. As we were especially interested in the top-down regulation of psychopathology we did not include the interaction of BIS X BAS (the interplay between anxiety and reward). Our findings thus especially highlight the role of EC in the expression of psychopathology/comorbidity also in an adult sample of SUD patients of which is known they are already characterized by relatively low overall levels of self-control [60,66].

The literature shows that also in a non-clinical sample adults with poor EC were more likely to report a higher number and greater severity of psychiatric symptoms [67]. In studies of executive functioning (EF) [68,69], which is closely related to EC, there is evidence that impairments in cognitive control are related with almost all forms of psychopathology [68]. Several studies support the role of EC as a transdiagnostic dimension covering a continuum from normal mental health to psychiatric disorders/psychological problems cutting through the boundaries of both internalizing and externalizing disorders [9].

Therefore, knowing that EC plays a very important role in psychopathology, therapeutic interventions aiming to strengthen cognitive control/EC might result in better treatment outcomes in patients with SUD for both the addiction and comorbid psychopathology. Targeting the construct of EC in treatment may contribute to reductions across psychopathology and can be seen as a transdiagnostic approach which has the benefit to address several comorbidities at the same time. Cognitive-behavioral therapy (CBT) is the main method of psychotherapy generally accepted in the field of substance addiction and non-substance addiction and is designed to be applied to a variety of psychiatric disorders. Especially the “third wave therapies”. e.g., mindfulnes-based interventions, Acceptance and Commitment Therapy (ACT), Dialectic Behavioral Therapy (DBT) can be seen as transdiagnostic treatments [70]. Further, cognitive training, e.g., Cognitive Bias Modification and training of working memory, has recently been applied in the treatment of for example, SUDs, mood and anxiety disorders [17,71,72,73,74]. However, in spite of some promising hypotheses and a limited number of positive outcome studies, the findings on the effectivity of cognitive training modules remain inconsistent.

Although this study has some strengths, especially in the large sample studied, some limitations should also be noted. First, the cross-sectional design used in the current study does not allow to infer causality. Second, we did not have a control group without SUD nor compared between different substances. Future research should thus include a control group and/or compare between different substances. Third, we only used self-report measures to assess temperament and severity of psychopathology, which increases the possibility of shared method variance inflating associations between the study variables. Future studies should thus combine self-report questionnaires assessing temperament with behavioral tasks. Further it seems important to investigate the influence of “age of onset” and “duration” of the substance abuse as it is known that chronic substance use further undermines the efficiency of control networks weakening the capacity for self-regulation.

To address the last two limitations, in our next study we shall combine self-report questionnaires assessing EC and behavioral tasks using the Cambridge Neuropsychological Test Automated Battery (CANTAB) and take into account the age of onset and duration of substance abuse.

## 5. Conclusions

Taken together, we found that low EC is involved in all clinical symptomatology and PDs in a sample of inpatients with SUDs. These findings are consistent with conceptualizations of EC as a major psychological dimension that may play a transdiagnostic role in shaping the risk for psychopathology. Therapeutic interventions aiming at strengthening cognitive control/EC can possibly result in better treatment outcomes for both the addiction and the comorbid psychopathology.

## Figures and Tables

**Table 1:** Type of Substance Used and Gender.

	Alcohol	Alcohol and Other Substance	Other Substance	Total
Male	121	393	36	552
Female	56	190	19	265
Total	177	583	57	817

Other substance = cocaine, amphetamine, cannabis, benzodiazepine, opioid analgesic.

**Table 2:** Descriptive statistics of EC, BIS and BAS for the total sample.

	N	Minimum	Maximum	M	(*SD*)
Leeftijd	841	17	71	42.86	(11.74)
ECtot	800	1.68	6.53	4.31	(0.82)
BIS	835	7.00	28.00	20.93	(4.09)
BAStot	835	16.00	52.00	36.51	(6.54)

EC = Effortful Control; BIS = Behavioral Inhibition System; BAS = Behavioral Activation System.

**Table 3:** Hierarchical regression analyses with SCL-90 symptoms scales as dependent variables.

	AGO	ANX	DEP	HOS	IN	PSYCH	DIS	SLE	SOM	SCL_TOT
	β	β	β	β	β	β	β	β	β	β
GENDER	−0.050	−0.072 *	−0.072 *	−0.091 *	−0.034	−0.042	−0.029	−0.014	−0.060	−0.072 *
AGE	0.012	0.046	0.031	−0.099 **	0.017	0.024	−0.006	−0.016	0.096 **	0.025
	R^2^ = 0.00	R^2^ = 0.01 *	R^2^ = 0.01	R^2^ = 0.02 **	R^2^ = 0.00	R^2^ = 0.00	R^2^ = 0.00	R^2^ = 0.01 **	R^2^ = 0.01 **	R^2^ = 0.006
										
GENDER	0.005	−0.008	0.001	−0.052	0.040	0.025	0.032	−0.087 *	−0.006	0.004
AGE	0.027	0.069 *	0.048	−0.092 **	0.043	0.052	0.016	−0.018	0.103 **	0.047
EC	−0.344 ***	−0.374 ***	−0.337 ***	−0.424 ***	−0.436 ***	−0.421 ***	−0.373 ***	−0.153 ***	−0.229 ***	−0.430 ***
BIS	0.079 *	0.098 **	0.137 ***	0.003 **	0.115 ***	0.097 **	0.090 *	0.041	0.105	0.121 ***
BAS	−0.013	0.000	−0.034	−0.008	−0.004	0.013	−0.002	−0.035	−0.046	−0.019
	R^2^ = 0.13 ***	R^2^ = 0.16 ***	R^2^ = 0.14 ***	R^2^ = 0.19 ***	R^2^ = 0.21 ***	R^2^ = 0.19 ***	R^2^ = 0.15 ***	R^2^ = 0.04 ***	R^2^ = 0.08	R^2^ = 0.21 ***
										
GENDER	0.006	−0.007	0.001	−0.053	0.040	0.026	0.032	−0.087 *	−0.004	0.005
AGE	0.027	0.067 *	0.047	−0.091 **	0.043	0.051	0.015	−0.018	0.100 **	0.046
EC	−0.345 ***	−0.374 ***	−0.337 ***	−0.423 ***	−0.436 ***	−0.421 ***	−0.373 ***	−0.153 ***	−0.229 ***	−0.430 ***
BIS	0.080 *	0.098 **	0.136 ***	0.003 ***	0.015 ***	0.097 **	0.089 *	0.040	0.104 **	0.120 ***
BAS	−0.013	−0.001	−0.034	−0.008	−0.004	0.013	−0.002	−0.036	−0.046	−0.019
BIS × EC	−0.046	−0.001	0.009	0.016	0.010	-0.012	0.008	0.030	−0.008	0.002
BAS × EC	−0.005	0.040	0.022	−0.020	0.014	0.019	0.004	0.016	0.072	0.026
	R^2^ = 0.13 ***	R^2^ = 0.16 ***	R^2^ = 0.1 ***	R^2^ = 0.19 ***	R^2^ = 0.21 ***	R^2^ = 0.19 ***	R^2^ = 0.15 ***	R^2^ = 0.04 ***	R^2^ = 0.08 ***	R^2^ = 0.21 ***

AGO = agoraphobia; ANX = anxiety; DEP = depression; HOS = hostility; IN = insufficiency of thought and behavior; PSCYH = psychoticism dimension; DIS = distrust and interpersonal sensitivity; SLE = sleeping problems; SOM = somatization problems. * *p* < 0.05, ** *p* < 0.01, *** *p* < 0.001.

**Table 4:** Hierarchical regression analyses with ADP-IV PD scores as dependent variables.

	Cluster A PDs			Cluster B PDs				Cluster C PDs		
	PAR	SZ	ST	AS	BDL	HIS	NARC	AV	DEP	OC
	β	β	β	β	β	β	β	β	β	β
GENDER	−0.056	0.015	−0.040	−0.053	−0.117 ***	−0.089 *	−0.036	−0.030	−0.075 *	−0.063
AGE	−0.095 **	−0.037	−0.049	−0.114 **	−0.073 *	−0.084 *	−0.079 *	−0.023	−0.017	−0.053
	R^2^ = 0.01 *	R^2^ = 0.00	R^2^ = 0.00	R^2^ = 0.01 *	R^2^ = 0.02 *	R^2^ = 0.01 **	R^2^ = 0.01	R^2^ = 0.00	R^2^ = 0.01	R^2^ = 0.01
										
GENDER	−0.027	0.052	0.007	−0.021	−0.060 *	−0.030	−0.017	0.030	−0.013	−0.017
AGE	−0.073 *	−0.028	−0.032	−0.092 **	−0.046	−0.046	−0.049	−0.008	0.006	−0.034
EC	−0.0335 ***	−0.156 ***	−0.421 ***	−0.539 ***	−0.548 ***	−0.532 ***	−0.366 ***	−0.386 ***	−0.469 ***	−0.200 ***
BIS	0.000	0.076 *	0.036	−0.041	0.036	0.051	−0.032	0.086 *	0.071 *	0.093 **
BAS	0.048	−0.017	0.011	0.054	0.033	0.064 *	0.088 *	−0.020	0.010	0.003
	R^2^ = 0.14 ***^a^	R^2^ = 0.03 ***	R^2^ = 0.18 ***^a^	R^2^ = 0.30 ***	R^2^ = 0.32 ***	R^2^ = 0.30 ***	R^2^ = 0.15 ***	R^2^ = 0.16 ***	R^2^ = 0.23 ***	R^2^ = 0.06 ***
										
GENDER	−0.029	0.055	0.009	−0.021	−0.061 *	−0.029	−0.016	0.029	−0.015	−0.018
AGE	−0.072 *	−0.029	−0.033	−0.092 **	−0.045	−0.047	−0.049	−0.008	0.007	−0.036
EC	−0.035 ***	−0.158 ***	−0.422 ***	−0.539 ***	−0.547 ***	−0.533 ***	−0.366 ***	−0.386 ***	−0.468 ***	−0.199 ***
BIS	0.000	0.077 *	0.037	−0.040	0.035	0.052	−0.030	0.086 **	0.070 **	0.091 **
BAS	0.049	−0.017	0.011	0.055	0.033	0.084 *	0.089 **	−0.020	0.010	0.002
BIS × EC	0.011	−0.069 *	−0.044	−0.014	0.031	−0.032	−0.044	0.012	0.034	0.049
BAS × EC	−0.035	0.028	0.014	−0.016	−0.013	0.010	−0.010	−0.023	−0.014	0.046
	R^2^ = 0.14 ***	R^2^ = 0.04 ***	R^2^ = 0.1 ***	R^2^ = 0.30 ***	R^2^ = 0.32 ***	R^2^ = 0.30 ***	R^2^ = 0.15 ***	R^2^ = 0.16 ***	R^2^ = 0.23 ***	R^2^ = 0.06 ***

PAR, paranoid PD; SZ, schizoid PD; ST, schizotypal PD; AS, antisocial PD; BDL, borderline PD; HIS, histrionic PD; NARC, narcissistic PD; AV, avoidant PD: DEP, dependent PD; OC, obsessive compulsive PD. ^a^ A significant increase in R^2^ compared to prior step. * *p* < 0.05, ** *p* < 0.01, *** *p* < 0.001.

## Data Availability

Data are available from the authors upon request.

## Supplementary Materials

The following supporting information can be downloaded at: https://www.mdpi.com/article/10.3390/jcm11030591/s1, Table S1: Type of Substance Used and Gender (12 categories).
