# Supplementary material for: Reactive and Regulative Temperament in Relation to Clinical Symptomatology and Personality Disorders in Patients with a Substance Use Disorder
Source: J Clin Med. 2022 Jan 25;11(3):591. doi: 10.3390/jcm11030591 (PMC8837055; doi:10.3390/jcm11030591)
Supplement: Supplementary file 1 [file jcm-11-00591-s001.zip › jcm-1474377-supplementary.pdf]

Journal of
Clinical Medicine

MDPI

Supplemental Material

Table S1. Type of Substance Used and Gender (12 categories).

	Male	Female	Total
Alcohol	121	56	177
Cocaine	7	3	11
Cannabis	4	1	5
Benzodiazepines (BZD)	17	11	26
Alcohol+ BZD	141	101	242
Alcohol+opioidanalgesics	27	19	41
Alcohol +cannabis	19	5	24
Polysubstance	199	69	268
Alcohol + cocaine	11	1	2
Alcohol + amfetamine	4	0	12
Methadon	1	0	1
Opioid analgesics	1	0	24
Total	552	265	817
